# Probability, clinical decision making and hypothesis testing

**DOI:** 10.4103/0972-6748.57864

**Published:** 2009

**Authors:** A. Banerjee, S. L. Jadhav, J. S. Bhawalkar

**Affiliations:** Department of Community Medicine, D. Y. Patil Medical College, Pune - 411018, India

**Keywords:** Hypothesis testing, *P* value, Probability

## Abstract

Few clinicians grasp the true concept of probability expressed in the ‘*P* value.’ For most, a statistically significant *P* value is the end of the search for truth. In fact, the opposite is the case. The present paper attempts to put the *P* value in proper perspective by explaining different types of probabilities, their role in clinical decision making, medical research and hypothesis testing.

The clinician who wishes to remain abreast with the results of medical research needs to develop a statistical sense. He reads a number of journal articles; and constantly, he must ask questions such as, “Am I convinced that lack of mental activity predisposes to Alzheimer’s? Or “Do I believe that a particular drug cures more patients than the drug I use currently?”

The results of most studies are quantitative; and in earlier times, the reader made up his mind whether or not to accept the results of a particular study by merely looking at the figures. For instance, if 25 out of 30 patients were cured with a new drug compared with 15 out of the 30 on placebo, the superiority of the new drug was readily accepted.

In recent years, the presentation of medical research has undergone much transformation. Nowadays, no respectable journal will accept a paper if the results have not been subjected to statistical significance tests. The use of statistics has accelerated with the ready availability of statistical software. It has now become fashionable to organize workshops on research methodology and biostatistics. No doubt, this development was long overdue and one concedes that the methodologies of most medical papers have considerably improved in recent years. But at the same time, a new problem has arisen. The reading of medical journals today presupposes considerable statistical knowledge; however, those doctors who are not familiar with statistical theory tend to interpret the results of significance tests uncritically or even incorrectly.

It is often overlooked that the results of a statistical test depend not only on the observed data but also on the choice of statistical model. The statistician doing analysis of the data has a choice between several tests which are based on different models and assumptions. Unfortunately, many research workers who know little about statistics leave the statistical analysis to statisticians who know little about medicine; and the end result may well be a series of meaningless calculations.

Many readers of medical journals do not know the correct interpretation of ‘*P* values,’ which are the results of significance tests. Usually, it is only stated whether the *P* value is below 5% (*P*< .05) or above 5% (*P* > .05). According to convention, the results of *P* < .05 are said to be statistically significant, and those with *P* > .05 are said to be statistically nonsignificant. These expressions are taken so seriously by most that it is almost considered ‘unscientific’ to believe in a nonsignificant result or not to believe in a ‘significant’ result. It is taken for granted that a ‘significant’ difference is a true difference and that a ‘nonsignificant’ difference is a chance finding and does not merit further exploration. Nothing can be further from the truth.

The present paper endeavors to explain the meaning of probability, its role in everyday clinical practice and the concepts behind hypothesis testing.

## WHAT IS PROBABILITY?

Probability is a recurring theme in medical practice. No doctor who returns home from a busy day at the hospital is spared the nagging feeling that some of his diagnoses may turn out to be wrong, or some of his treatments may not lead to the expected cure. Encountering the unexpected is an occupational hazard in clinical practice. Doctors after some experience in their profession reconcile to the fact that diagnosis and prognosis always have varying degrees of uncertainty and at best can be stated as *probable* in a particular case.

Critical appraisal of medical journals also leads to the same gut feeling. One is bombarded with new research results, but experience dictates that well-established facts of today may be refuted in some other scientific publication in the following weeks or months. When a practicing clinician reads that some new treatment is superior to the conventional one, he will assess the evidence critically, and at best he will conclude that *probably* it is true.

### Two types of probabilities

The statistical probability concept is so widely prevalent that almost everyone believes that probability is a *frequency*. It is not, of course, an ordinary frequency which can be estimated by simple observations, but it is the *ideal* or *truth in the universe*, which is reflected by the observed frequency. For example, when we want to determine the probability of obtaining an ace from a pack of cards (which, let us assume has been tampered with by a dishonest gambler), we proceed by drawing a card from the pack a large number of times, as we know in the long run, the observed frequency will approach the true probability or truth in the universe. Mathematicians often state that a probability is a long-run frequency, and a probability that is defined in this way is called a *frequential probability*. The exact magnitude of a frequential probability will remain elusive as we cannot make an infinite number of observations; but when we have made a decent number of observations (adequate sample size), we can calculate the confidence intervals, which are likely to include the true frequential probability. The width of the confidence interval depends on the number of observations (sample size).

The frequential probability concept is so prevalent that we tend to overlook terms like chance, risk and odds, in which the term *probability* implies a different meaning. Few hypothetical examples will make this clear. Consider the statement, “The cure for Alzheimer’s disease will *probably* be discovered in the coming decade.” This statement does not indicate the basis of this expectation or belief as in frequential probability, where a number of repeated observations provide the foundation for probability calculation. However, it may be based on the present state of research in Alzheimer’s. A probabilistic statement incorporates some amount of uncertainty, which may be quantified as follows: A politician may state that there is a *fifty-fifty* chance of winning the next election, a bookie may say that the *odds* of India winning the next one-day cricket game is four to one, and so on. At first glance, such probabilities may appear frequential ones, but a little reflection will reveal the contrary. We are concerned with unique events, i.e., the likely cure of a disease in the future, the next particular election, the next particular one-day game — and it makes no sense to apply the statistical idea that these types of probabilities are long-run frequencies. Further reflection will illustrate that these statements about probabilities of the election and one-day game are no different from the one about the cure for Alzheimer’s, apart from the fact that in the latter cases an attempt has been made to quantify the magnitude of belief in the occurrence of the event.

It follows from the above deliberations that we have 2 types of probability concepts. In the jargon of statistics, a probability is ideal or truth in the universe which lies beneath an observed frequency — such probabilities may be called frequential probabilities. In literary language, a probability is a measure of our subjective belief in the occurrence of a particular event or truth of a hypothesis. Such probabilities, which may be quantified that they look like frequential ones, are called subjective probabilities. Bayesian statistical theory also takes into account subjective probabilities (Lindley, 1973; Winkler, 1972). The following examples will try to illustrate these (rather confusing) concepts.

A young man is brought to the psychiatry OPD with history of withdrawal. He also gives history of talking to himself and giggling without cause. There is also a positive family history of schizophrenia. The consulting psychiatrist who examines the patient concludes that there is a 90% probability that this patient suffers from schizophrenia.

We ask the psychiatrist what makes him make such a statement. He may not be able to say that he knows from experience that 90% of such patients suffer from schizophrenia. The statement therefore may not be based on observed frequency. Instead, the psychiatrist states his probability based on his knowledge of the natural history of disease and the available literature regarding signs and symptoms in schizophrenia and positive family history. From this knowledge, the psychiatrist concludes that his belief in the diagnosis of schizophrenia in that particular patient is as strong as his belief in picking a black ball from a box containing 10 white and 90 black balls. The probability in this case is certainly *subjective probability*.

Let us consider another example: A 26-year-old married female patient who suffered from severe abdominal pain is referred to a hospital. She is also having amenorrhea for the past 4 months. The pain is located in the left lower abdomen. The gynecologist who examines her concludes that there is a 30% probability that the patient is suffering from ectopic pregnancy.

As before, we ask the gynecologist to explain on what basis the diagnosis of ectopic pregnancy is suspected. In this case the gynecologist states that he has studied a large number of successive patients with this symptom complex of lower abdominal pain with amenorrhea, and that a subsequent laparotomy revealed an ectopic pregnancy in 30% of the cases.

If we accept that the study cited is large enough to make us assume that the possibility of the observed frequency of ectopic pregnancy did not differ from the true frequential probability, it is natural to conclude that the gynecologist’s probability claim is more ‘evidence based’ than that of the psychiatrist, but again this is debatable.

In order to grasp this in proper perspective, it is necessary to note that the gynecologist stated that the probability of ectopic pregnancy in that *particular patient* was 30%. Therefore, we are concerned with a unique event just as the politician’s next election or India’s next one-day match. So in this case also, the probability is a *subjective probability which was based on an observed frequency*. One might also argue that even this probability is not good enough. We might ask the gynecologist to base his belief on a group of patients who also had the same age, height, color of hair and social background; and in the end, the reference group would be so restrictive that even the experience from a very large study would not provide the necessary information. If we went even further and required that he must base his belief on patients who in all respects resembled this particular patient, the probabilistic problem would vanish as we will be dealing with a certainty rather than a probability.

The clinician’s belief in a particular diagnosis in an individual patient may be based on the recorded experience in a group of patients, but it is still a subjective probability. It reflects not only the observed frequency of the disease in a reference group but also the clinician’s theoretical knowledge which determines the choice of reference group (Wulff, Pedersen and Rosenberg, 1986). Recorded experience is never the sole basis of clinical decision making.

## GAP BETWEEN THEORY AND PRACTICE

The two situations described above are relatively straightforward. The physician observed a patient with a particular set of signs and symptoms and assessed the subjective probability about the diagnosis in each case. Such probabilities have been termed *diagnostic probabilities* (Wulff, Pedersen and Rosenberg, 1986). In practice, however, clinicians make diagnosis in a more complex manner which they themselves may be unable to analyze logically.

For instance, suppose the clinician suspects one of his patients is suffering from a rare disease named ‘D.’ He requests a suitable test to confirm the diagnosis, and suppose the test is positive for disease ‘D.’ He now wishes to assess the probability of the diagnosis being positive on the basis of this information, but perhaps the medical literature only provides the information that a positive test is seen in 70% of the patients with disease ‘D.’ However, it is also positive in 2% of patients without disease ‘D.’ How to tackle this doctor’s dilemma? First a formal analysis may be attempted, and then we can return to everyday clinical thinking. The frequential probability which the doctor found in the literature may be written in the statistical notation as follows:

*P*(S/D+) = .70, i.e., the probability of the presence of this particular sign (or test) given this particular disease is 70%.

*P*(S/D–) = .02, i.e., the probability of this particular sign given the absence of this particular disease is 2%.

However, such probabilities are of little clinical relevance. The clinical relevance is in the ‘opposite’ probability. In clinical practice, one would like to know the *P*(D/S), i.e., the probability of the disease in a particular patient given this positive sign. This can be estimated by means of Bayes’ Theorem (Papoulis, 1984; Lindley, 1973; Winkler, 1972). The formula of Bayes’ Theorem is reproduced below, from which it will be evident that to calculate P(D/S), we must also know the prior probability of the presence and the absence of the disease, i.e., *P*(D+) and *P*(D–).

*P*(D/S) = *P*(S/D+) *P*(D+) ÷ *P*(S/D+) *P*(D+) + *P*(S/D–) *P*(D–)

In the example of the disease ‘D’ above, let us assume that we estimate that prior probability of the disease being present, i.e., *P* (D+), is 25%; and therefore, prior probability of the absence of disease, i.e., *P* (D–), is 75%. Using the Bayes’ Theorem formula, we can calculate that the probability of the disease given a positive sign, i.e., *P*(D/S), is 92%.

We of course do not suggest that clinicians should always make calculations of this sort when confronted with a diagnostic dilemma, but they must in an intuitive way think along these lines. Clinical knowledge is to a large extent based on textbook knowledge, and ordinary textbooks do not tell the reader much about the probabilities of different diseases given different symptoms. Bayes’ Theorem guides a clinician how to use textbook knowledge for practical clinical purposes.

The practical significance of this point is illustrated by the European doctor who accepted a position at a hospital in tropical Africa. In order to prepare himself for the new job, he bought himself a large textbook of tropical medicine and studied in great detail the clinical pictures of a large number of exotic diseases. However, for several months after his arrival at the tropical hospital, his diagnostic performance was very poor, as he knew nothing about the relative frequency of all these diseases. He had to acquaint himself with the prior probability, *P* (D +), of the diseases in the catchment area of the hospital before he could make precise diagnoses.

The same thing happens on a smaller scale when a doctor trained at a university hospital establishes himself in general practice. At the beginning, he will suspect his patients of all sorts of rare diseases (which are common at the university hospital), but after a while he will learn to assess correctly the frequency of different diseases in the general population.

## PROBABILITY AND HYPOTHESIS TESTING

Besides predictions on individual patients, the doctor is also concerned in generalizations to the population at large or the target population. We may say that probably there may have been life at Mars. We may even quantify our belief and mention that there is 95% probability that depression responds more quickly during treatment with a particular antidepressant than during treatment with a placebo. These probabilities are again *subjective probabilities* rather than *frequential probabilities*. The last statement does not imply that 95% of depression cases respond to the particular antidepressant or that 95% of the published reports mention that the particular antidepressant is the best. It simply means that our belief in the truth of the statement is the same as our belief in picking up a red ball from a box containing 95 red balls and 5 white balls. It means that we are, however, almost not totally convinced that the average recovery time during treatment with a particular antidepressant is shorter than during placebo treatment.

The purpose of hypothesis testing is to aid the clinician in reaching a conclusion concerning the universe by examining a sample from that universe. A hypothesis may be defined as a presumption or statement about the truth in the universe. For example, a clinician may hypothesize that a certain drug may be effective in 80% of the cases of schizophrenia. It is frequently concerned about the parameters in the population about which the presumption or statement is made. It is the basis for motivating the research project. There are two types of hypotheses, research hypothesis and statistical hypothesis (Daniel, 2000; Guyatt *et al*., 1995).

### Genesis of research hypothesis

Hypothesis may be generated by deduction from anatomical, physiological facts or from clinical observations.

### Statistical hypothesis

Statistical hypotheses are hypotheses that are stated in such a way that they may be evaluated by appropriate statistical techniques.

### Pre-requisites for hypothesis testing

#### Nature of data

The types of data that form the basis of hypothesis testing procedures must be understood, since these dictate the choice of statistical test.

#### Presumptions

These presumptions are the normality of the population distribution, equality of the standard deviations, random samples.

#### Hypothesis

There are 2 statistical hypotheses involved in hypothesis testing. These should be stated a *priori* and explicitly. The *null hypothesis* is the hypothesis to be tested. It is denoted by the symbol *H*_0_. It is also known as *the hypothesis of no difference*. The null hypothesis is set up with the sole purpose of efforts to knock it down. In the testing of hypothesis, the null hypothesis is either *rejected* (knocked down) or *not rejected* (upheld). If the null hypothesis is not rejected, the interpretation is that the data is not sufficient evidence to cause rejection. If the testing process rejects the null hypothesis, the inference is that the data available to us is not compatible with the null hypothesis and by default we accept the *alternative hypothesis*, which in most cases is the research hypothesis. The *alternative hypothesis* is designated with the symbol *H*_A_.

#### Limitations

Neither hypothesis testing nor statistical tests lead to proof. It merely indicates whether the hypothesis is supported or not supported by the available data. When we reject a null hypothesis, we do not mean it is not true but that it may be true. By default when we do not reject the null hypothesis, we should have this limitation in mind and should not convey the impression that this implies proof.

#### Test statistic

The test statistic is the statistic that is derived from the data from the sample. Evidently, many possible values of the test statistic can be computed depending on the particular sample selected. The test statistic serves as a decision maker, nothing more, nothing less, rather than proof or lack of it. The decision to reject or not to reject the null hypothesis depends on the magnitude of the test statistic.

#### Types of decision errors

The error committed when a true null hypothesis is rejected is called the type *I error or α error*. When a false null hypothesis is not rejected, we commit type *II error, or β error*. When we reject a null hypothesis, there is always the risk (howsoever small it may be) of committing a type I error, i.e., rejecting a true null hypothesis. On the other hand, whenever we fail to reject a null hypothesis, the risk of failing to reject a false null hypothesis, or committing a type II error, will always be present. Put in other words, the test statistic does not eliminate uncertainty (as many tend to believe); it only quantifies our uncertainty.

#### Calculation of test statistic

From the data contained in the sample, we compute a value of the test statistic and compare it with the rejection and non-rejection regions, which have to be specified in advance.

#### Statistical decision

The statistical decision consists of rejecting or of not rejecting the null hypothesis. It is rejected if the computed value of the test statistic falls in the rejection region, and it is not rejected if the value falls in the non-rejection region.

#### Conclusion

If H_0_ is rejected, we conclude that H_A_ is true. If H_0_ is not rejected, we conclude that H_0_ may be true.

#### P values

The *P* value is a number that tells us how unlikely our sample values are, given that the null hypothesis is true. A *P* value indicating that the sample results are not likely to have occurred, if the null hypothesis is true, provides reason for doubting the truth of the null hypothesis.

We must remember that, when the null hypothesis is not rejected, one should not say the null hypothesis is accepted. We should mention that the null hypothesis is “not rejected.” We avoid using the word *accepted* in this case because we may have committed a type II error. Since, frequently, the probability of committing error can be quite high (particularly with small sample sizes), we should not commit ourselves to accepting the null hypothesis.

## INTERPRETATIONS

With the above discussion on probability, clinical decision making and hypothesis testing in mind, let us reconsider the meaning of *P* values. When we come across the statement that there is statistically significant difference between two treatment regimes with *P* < .05, we should not interpret that there is less than 5% probability that there is no difference, and that there is 95% probability that a difference exists, as many uninformed readers tend to do. The statement that there is difference between the cure rates of two treatments is a general one, and we have already discussed that the probability of the truth of a general statement (hypothesis) is *subjective*, whereas the probabilities which are calculated by statisticians are frequential ones. The hypothesis that one treatment is better than the other is either true or false and cannot be interpreted in frequential terms.

To explain this further, suppose someone claims that 20 (80%) of 25 patients who received drug A were cured, compared to 12 (48%) of 25 patients who received drug B. In this case, there are two possibilities, either the *null hypothesis* is true, which means that the two treatments are equally effective and the observed difference arose by chance; or the *null hypothesis* is not true (and we accept the *alternative hypothesis* by default), which means that one treatment is better than the other. The clinician wants to make up his mind to what extent he believes in the truth of the *alternative hypothesis* (or the *falsehood of the null hypothesis*). To resolve this issue, he needs the aid of statistical analysis. However, it is essential to note that the *P* value does not provide a direct answer. Let us assume in this case the statistician does a significance test and gets a *P* value = .04, meaning that the difference is statistically significant (*P* < .05). But as explained earlier, this does not mean that there is a 4% probability that the null hypothesis is true and 96% chance that the alternative hypothesis is true. The *P* value is a frequential probability and it provides the information that there is a 4% probability of obtaining such a difference between the cure rates, *if the null hypothesis is true*. In other words, the statistician asks us to assume that the null hypothesis is true and to imagine that we do a large number of trials. In that case, the long-run frequency of trials which show a difference between the cure rates like the one we found, or even a larger one, will be 4%.

### Prior belief and interpretation of the *P* value

In order to elucidate the implications of the correct statistical definition of the *P* value, let us imagine that the patients who took part in the above trial suffered from depression, and that drug A was gentamycin, while drug B was a placebo. Our theoretical knowledge gives us no grounds for believing that gentamycin has any affect whatsoever in the cure of depression. For this reason, our prior confidence in the truth of the null hypothesis is immense (say, 99.99%), whereas our prior confidence in the alternative hypothesis is minute (0.01%). We must take these prior probabilities into account when we assess the result of the trial. We have the following choice. Either we accept the null hypothesis in spite of the fact that the probability of the trial result is fairly low at 4% (*P* < .05) given the null hypothesis is true, or we accept the alternative hypothesis by rejecting the null hypothesis in spite of the fact that the *subjective* probability of that hypothesis is extremely low in the light of our prior knowledge.

It will be evident that the choice is a difficult one, as both hypotheses, each in its own way, may be said to be unlikely, but any clinician who reasons along these lines will choose that hypothesis which is least unacceptable: He will accept the null hypothesis and claim that the difference between the cure rates arose by chance (however small it may be), as he does not feel that the evidence from this single trial is sufficient to shake his prior belief in the null hypothesis.

## CONCLUSION

Misinterpretation of *P* values is extremely common. One of the reasons may be that those who teach research methods do not themselves appreciate the problem. The *P* value is the probability of obtaining a value of the test statistic as large as or larger than the one computed from the data when in reality there is no difference between the different treatments. In other words, the *P* value is the probability of being wrong when asserting that a difference exists.

Lastly, we must remember we do not establish proof by hypothesis testing, and uncertainty will always remain in empirical research; at the most, we can only quantify our uncertainty.
